# The lancet series nutritional interventions in Ghana: a determinants analysis approach to inform nutrition strategic planning

**DOI:** 10.1186/s40795-017-0147-1

**Published:** 2017-03-15

**Authors:** A.E. Yawson, E.O. Amoaful, L.K. Senaya, A.O. Yawson, P.K. Aboagye, A.B. Mahama, L. Selenje, V. Ngongalah

**Affiliations:** 1 Department of Community Health, School of Public Health, College of Health Sciences, University of Ghana, Room 46, P. O. Box 4236, Accra, Ghana; 2 Family Health Division, Ghana Health Service, Accra, Ghana; 3 New Juabeng Municipal Health Directorate, Ghana Health Service, Koforidua, Ghana; 4 Department of Child Health, Korle-Bu Teaching Hospital, Accra, Ghana; 5 UNICEF Country Office, Accra, Ghana

**Keywords:** Nutrition, Children, Determinants Analysis, Health planning, Ghana

## Abstract

**Background:**

Malnutrition is a leading cause of mortality and morbidity among children in low- and middle-income countries. Ghana is one of 36 countries with the highest burden of stunting, globally. The aim of this work is to use data driven planning methods to conduct in-depth analysis on the Lancet series nutrition interventions in Ghana to inform nutritional strategic planning.

**Methods:**

A mixed methods approach was employed for this national nutritional assessment conducted in May 2016. Quantitative data on nutritional interventions were generated by application of the Determinants Analysis Tool and phenomenological approach was employed to explain the causes of barriers identified. Outputs from the tool were analyzed by simple descriptive statistics and data from group discussions were assessed by thematic content analysis. The base line years for this assessment were 2014 and 2015.

**Results:**

Overall in Ghana, 21.0% of frontline health workers are trained on lactation management and breastfeeding counselling and support, 56.6% of mothers of children 0–2 years initiated breastfeeding within one hour of birth, and 59.4% of mothers with children 0–5 months took iron folate supplementation for 90 or more days during pregnancy. In addition, only 19.9% of children 12–59 months received two doses of vitamin A supplementation in a calendar year, and 32.5% of children 6–59 months with severe acute malnutrition were admitted for treatment at health facilities. In all, among infants 6–8 months old, 6.9% were fed with minimum dietary diversity, 50.6% received age appropriate meal frequency and 21.6% received iron rich diet. Inadequate pre-service and in-service training for staff, low prioritization and coordination (at higher levels) and weak integration of services (at lower levels) were key barriers to nutrition coverage in Ghana.

**Conclusion:**

Data driven analysis and planning based on proven nutritional interventions in Ghana demonstrated gaps and barriers and garnered workable strategies to improve nutrition services.

## Background

Malnutrition is a leading cause of mortality and morbidity among children in low- and middle-income countries [1, 2]. In Sub-Saharan Africa, under nutrition constitutes a leading cause of death in children under five years old [3, 4]. Multiple interventions and multi-sectoral approach are needed to prevent different aspects of nutritional depletion and deprivation such as severe malnutrition, anaemia, wasting and stunting [2, 5, 6].

The implications and consequences of these nutritional deprivation in later adult life make it imperative for national nutritional programmes to focus on sufficient feeding for individuals, families and communities as well as a responsive health care delivery system [3, 7, 8]. Child malnutrition within a household is greatly influenced by issues at national and regional levels [9].

Ghana is beset with under nutrition among children, and is one of 36 countries with the highest burden of stunting, globally [4]. In Ghana 19% of children are stunted, 5% of children are wasted and 11% of children are underweight [10]. Anaemia as a result of under nutrition among children is another key national challenge, 66% of children aged 6–59 months have some level of anaemia, 37% have moderate levels of anaemia and 2% have severe anaemia [10, 11]. National coverage on interventions is relatively low with inadequate engagement of the health sector and other sectors to address food security, food safety and hygiene for sustained improvement [12].

Current national efforts to reduce nutritional deprivations in Ghana include building capacity of service providers and volunteers in nutritional counselling, and improving knowledge and skill of service providers in the management of severe acute malnutrition [12, 13]. In addition, national scale up of infant and young child feeding activities include strengthening health care practices through the Baby-Friendly Hospital Initiative and creating demand for services through participation of local leaders and communities on sociocultural practices around malnutrition [12, 13].

Progress has been made over the past decade, as demonstrated in the 2014 Demographic and Health Survey (GDHS) [10]. Infant mortality declined from 77 deaths per 1,000 live births in 1988 to 41 in 2014 and under-5 mortality declined substantially from 155 to 60 death per 1,000 live births over the same period. These improvements at the national level notwithstanding, inequities exist in status of children by regional and geographical location in the country [10, 11]. Under-5 mortality in 2014 ranged from 47 deaths per 1,000 live births in Greater Accra (the capital region) to 111 deaths per 1,000 live births in Northern region (a predominantly rural region), while stunting ranged from 10.4% in Greater Accra to 33.1% in the Northern Region. The improvements in anaemia, stunting and wasting among children in Ghana have generally been uneven, with wide geographical disparities and among different wealth quintiles [10].

Interestingly, the burden of nutrition in Ghana is not limited to children because adult men and women are affected as well. In adults under- and over nutrition are both demonstrated through national surveys to be a health challenge. Overall, among men and women aged 15–49 years, 6% of women and 10% of men are underweight (Body Mass Index, BMI <18.5), while 40% of women and 16% of men are overweight (BMI > =25.0) [10].

Major causes and determinants of malnutrition in Ghana include, inadequate diet diversity (insufficient food/nutrient intake and inadequate consumption of foods rich in micronutrients and protein including eggs and legumes); poor environmental, household and individual hygiene including hand hygiene practices; as well as generally poor infant and young child feeding practices [12].

This assessment of national nutrition interventions based on The Lancet Series-Maternal and Child Nutrition [14, 15] was thus intended to determine the status of these proven nutritional interventions in Ghana, identify barriers to service provision and develop workable strategies. It used data driven planning methods to conduct in-depth analysis needed to guide programme scale up and targeting, to improve coverage and scope of proven nutritional interventions.

## Methods

Mixed methods approach were employed for this national nutritional assessment conducted in May 2016. Quantitative data on nutritional interventions was generated by application of the Determinants Analysis Tool and phenomenological approach was employed to explain the causes of barriers identified. Four participants (Regional Deputy Director of Public Health, Regional Public Health Nurse, Regional Nutrition Officer and Regional Health Information Officer) were purposively selected from each of the ten administrative regions in the Ghana due to their background and role in nutrition services. In addition local facilitators (Nutrition Unit of the Ghana Health Service) and representatives from some development partners (including UNICEF, WHO and World Food Programme (WFP) participated. Ghana has ten administrative regions, and officers from all ten regions were included to provide a nationwide representation. The national assessment was undertaken in the Ashanti Region of Ghana for three days to assess barriers to coverage of nutrition services and included desk review, group discussions and key informants interviews. Facilitators assisted in the desk reviews, conduct of interviews and provided mentoring and coaching on capturing major issues and recording of key points from the group discussion.

Main causes of identified bottlenecks to the nutritional interventions were thematically analysed through a phenomenological approach under service delivery factors and enabling environment factors through group discussions. Phenomenological approach was used to allow a deeper understanding of an event or phenomenon by looking at the story of the group who experienced a shared lived experience or phenomenon [16]. Six teams made up of regional, national and international personnel were formed in line with the key interventions to provide qualitative explanation and assessment of the outputs from the tool.

### The determinants analysis framework

**Fig. 1:**
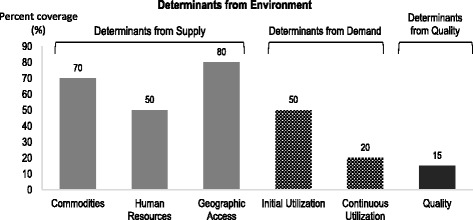
Graphical presentation of the determinants analysis framework. Description of figure: The figure describes the supply side, demand side and effective coverage determinants and how bottlenecks are determined

The Determinants Analysis framework is premised on the notion that effective coverage of services is influenced by four main factors or determinants namely: supply, demand, quality and environment. The Ten Determinant Model Tool produces a graphical output (Fig. 1) that facilitates identification of the key bottlenecks.

Overall, the Ten Determinant Model used for this nutrition assessment in Ghana had FOUR Domains with TEN Determinants: Enabling Environment (Social Norms, Management/Coordination, Legislation/Policy and Budget/Expenditures); Supply (Commodities, Human Resource and Access); Demand (Initial Utilisation, Continuous Utilisation/Knowledge) and Effective Coverage/Behaviour [17, 18].

Identification of the low bars on the supply-determinant side of the graph as well as a sharp drop from one bar to the next on the demand and quality-determinant side of the graph enables managers to identify and prioritize bottlenecks to the effective health service coverage.

### Data collection

The Ten Determinant Model is based on Microsoft Excel sheets linked together with sections for data inputs linked to outputs. Input data produce simple bar graphs that display the determinants of health service coverage and thus facilitate identification of key bottlenecks that influence effective coverage (illustrated in Fig. 1). The section for data input is fully linked with the section that displays the outputs. The tool directly utilised data from District Health Information Management System (DHIMS), Multi-Indicator Cluster Survey (MICS) Reports [19], Demographic and Health Surveys (DHS) [20] and other sources of data including programme monitoring and review reports. The DHIMS is a tool for collection, validation, analysis, and presentation of aggregate statistical data, tailored to integrated health information management activities in the Ghana Health Service.

### Outcome measures and data analysis

Six key nutrition tracer interventions based on The Lancet Series-Maternal and Child Nutrition [15], were selected as, Community *Management of Acute Malnutrition (CMAM), Complementary Feeding, Early Initiation to Breastfeeding, Exclusive Breastfeeding, Vitamin A Supplementation, and Iron-Folic Acid Supplementation*. Each intervention was assessed based on a particular delivery platform (as individualized facility based services or schedulable and outreach services or as family oriented practices and community based services) to enable accurate identification of barriers and bottlenecks. In this analysis, minimum acceptable diet for complementary feeding was assessed using two sub components-minimum dietary diversity and age appropriate meal frequency as specified in the Ministry of Health National Nutrition Policy of Ghana [12]. Each of the six thematic groups had a moderator and a recorder. Data from group discussions were organized and described using manual thematic content analysis for qualitative data. Each group worked on a different theme to eliminated the issue of duplication and repetition of information among the groups. Quantitative data were analyzed with simple descriptive statistics (i.e. proportions and percentages) by Microsoft Excel 2013.

The base line years for this assessment were 2014 and 2015 and national coverage targets were used as the benchmark to assess these coverage indicators. Outputs from the tool were analyzed by simple descriptive statistics such as frequency, proportions and ratios. The determinants analysis principles were applied to identify bottlenecks. Causality analysis were conducted to identify barriers resulting in these bottleneck. This formed the basis in determining strategies to remove the bottlenecks on nutritional deprivation in Ghana.

### Ethical Issues

All data used was aggregated data at national level and had no link to individuals. In all cases, documentations and computerized records were kept secure and accessible to persons directly involved in developing the operational plans. The Ghana Health Service Ethics Review Committee and office of the Policy, Planning, Monitoring and Evaluation Division of the Ghana Health Service gave approval for documentation and dissemination of the Determinant Analysis process in Ghana.

## Results

The assessment provided quantitative values on the performance of each indicator from outputs of the tool, while qualitative explanation and assessment of identified bottlenecks was through causality analysis and group discussions. Details of the analysis are indicated in a comprehensive descriptive Table (Table 1).

**Table 1:** Nutritional interventions, key bottlenecks and major causes of nutritional deprivations in ghana based on determinant analysis

Determinant	Identified Bottleneck (quantitative assessment)	Main Causes of Bottleneck (qualitative assessment)
***Early Initiation of Breastfeeding***	i. **Human resources:** Only 21.0% of health workers in health facilities are trained on lactation management/breastfeeding counselling and support ii. **Effective coverage:** Only 56.6% of mothers of children 0–2 years who gave birth in a health facility breastfed their infants within 1 hour of birth	**Human Resource**: *Service Delivery Level*-Inadequate integration of nutrition and breastfeeding component into Maternal and newborn care training *Enabling Environment*- Inadequate pre-service training on breastfeeding for frontline workers **Effective coverage** *Service Delivery Level*- Lack of support to mothers to breastfeed in first hour of birth, Poor staff attitude, Inhibitory Social Norms on Feeding after delivery (example prohibition of giving colostrum to the baby, inclusion of water in diet of newborn and keeping babies indoors for the first seven days), as well as preference for early introduction of formula by mother *Enabling Environment*-Poor enforcement of baby friendly policy in health facilities
***Exclusive Breastfeeding***	i. **Human resources:** Only 21.0% of health workers in health facilities are trained on lactation management/breastfeeding counselling and support ii. **Effective coverage:** Only 52.3% of children 0–5 months were exclusively breast-fed	**Human resource** *Service Delivery Level*- Unstructured orientation on exclusive breastfeeding counselling of frontline worker e.g. community health workers (CHW) not prioritized by sub-national health management teams *Enabling Environment* – Inadequate pre-service training on breastfeeding, and too many competing priorities at the higher levels of health delivery (Ministry of Health level and health training institutions) **Effective coverage** *Service Delivery Level-* inadequate engagement of family members by frontline workers during counselling at antenatal, postnatal, child welfare clinics and home visits. *Enabling Environment-* ineffective planning and conduct of home visits (mismatch between time of home visits and availability of service recipients) as well as financial and logistic constraints for effective transportation
***Iron and Folate Supplementation (IFA)***	i. **Data availability-** was a major bottleneck for the iron folate supplementation Determinant ii. **Effective coverage:** Only 59.4% of mothers with children 0–5 months took IFA for 90 or more days during pregnancy	**Effective coverage** *Service Delivery Level-* Frontline staff at the Antenatal clinic staff do not prioritize counselling on iron and folate supplementation during service provision, as well as non-compliance of pregnant women on iron folate supplementation (due to fear of side effects) *Enabling Environment-* National level health managers have not prioritized inclusion of iron folate supplementation data in health information management systems nationally. In addition weak supervision of frontline staff affects delivery of this service
***Vitamin A Supplementation*** ***(VAS)***	i. **Initial Utilisation-** Only 22.0% of children 12–59 months received Vitamin A supplementation (VAS) in semester 1 ii. **Initial Utilisation-** Only 19.9% of children 12–59 months received VAS in semester 2 iii. **Effective coverage:** Only 19.9% of children 12–59 months received two doses of VAS in a calendar year	**Initial Utilisation and Effective Coverage** *Service Delivery Level-* Limited counseling on the importance of vitamin A from frontline workers to caregivers, inefficient planned preventive & maintenance schedule for vehicles/motorbikes for outreach services and general inadequate coordination of training plans for frontline staff by health managers *Enabling Environment* – Absence of a national comprehensive Vitamin A supplementation strategy to reach children 12–59 months in routine services, limited data capture on children 0–59 months provided vitamin A in health information management systems and limited supervision and appraisal of frontline health workers using Vitamin A supplementation indicators
***Treatment and Management- Community based management of acute malnutrition (CMAM)***	**Initial Utilisation-** Only 32.5% of children 6–59 months with severe acute malnutrition (SAM) admitted for treatment at health facilities	**Initial Utilisation:** *Service Delivery Level-* Limited active surveillance on severe acute malnutrition within the communities (due to complaints of high workload by health workers), and inimical sociocultural beliefs among caregivers (that the cause and treatment of severe acute malnutrition is spiritual). In addition, there is limited frequency and intensity as well as targeting of cases of severe acute malnutrition with social mobilization as well as limited service access for treatment *Enabling Environment* – Gap between policy and implementation of the policy on free treatment for Children less than 5 years with severe acute malnutrition, Ineffective service integration, coordination and management of community based management of acute malnutrition
***Complementary Feeding (CF)***	i. **Human resource:** Only 21% of community health workers trained on complementary feeding counselling, support and communication ii. **Effective coverage** ^*^ **:** (This was assessed for all the 3 components of minimum acceptable diet) a. Only 6.9% of children 6–8 months were fed with minimum dietary diversity (with ≥ 4 food groups) b. In all 50.6% of children 6–8 months received age appropriate meal frequency in the past 24 hours **c. ** Only 21.6% of children 6–8 months received iron rich diet in the past 24 hours	**Human resource** *Service Delivery Level*- Ineffective planning and management of pre-service and in-service training, high attrition of frontline staff due to transfers and movement for further education as well as poor targeting and selection of health workers for training. *Enabling Environment –* Limited priority and coordination at higher levels of the health care system on comprehensive infant and young child feeding and the content of curriculum of health training institutions do not adequately cater for the practical skill required by frontline workers to provide optimum services. **Effective coverage** *Service Delivery Level-* Unavailability of diverse foods sources and inequitable intra-household food distribution, as well as a generally low intake of high bioavailable iron-rich foods at the family level *Enabling Environment –* Limited resources for service provision, limited availability of affordable home fortified iron-rich products on the market in the country and inadequate inter-sectoral collaboration.

## Discussion

Maternal and child undernutrition, consisting of stunting, wasting, and deficiencies of essential vitamins and minerals, was the subject of a Series of papers in *The Lancet* in 2008 [4, 7, 14]. It was suggested then that, long-term consequences of undernutrition could be reduced through high and equitable coverage of proven nutrition interventions. Included in these were the nutrition specific interventions [4, 7, 14]. Lancet series have been in existence close to eight years and have provided guidance to countries in their quest to improve health and nutrition of children under five years of age and Ghana is no exception. Six of the key nutrition specific interventions were selected for assessment at the national level in Ghana.

The analysis readily highlighted gaps in service coverage quantitatively (from service and survey data) and provided a platform for qualitatively analyzing barriers and bottlenecks in the continuum of care for the newborn. These discussions included key service providers and policy makers with sufficient knowledge of the context of nutritional service delivery in Ghana.

The current national efforts to reduce nutritional deprivations in Ghana outlined in the National Nutrition Policy of 2013 include building capacity of service providers and volunteers in nutritional counselling, improving knowledge and skill of service providers in the management of severe acute malnutrition and a national scale up of infant and young child feeding activities by strengthening health care practices through the Baby-Friendly Hospital Initiative [12, 13]. In addition, a number of initiatives and frameworks have been developed and implemented by the national Ministry of Health to address the problem of high under five mortality including the Child Health policy, Millennium Accelerated Framework, Accelerated immunization with introduction of new and additional vaccines, as well as the Global Funded programmes for Malaria, Tuberculosis and HIV [12].

The challenge has been major gaps in access and utilization of these interventions and the reality however is that, most of these interventions and frameworks have been centrally developed with little attention paid to data driven approaches and causal analysis of barriers at the service delivery level.

To overcome these challenges, the current analysis based the national nutritional strategies on the framework developed by Bezanson and Isenman in 2010 [21] to provide guidance as Ghana implements the national nutrition policy. The ‘Framework for Action for Scaling Up Nutrition’ in 2010 was based on a broad collaborative effort of the World Bank, United Nations Children Fund (UNICEF), World Health Organization (WHO), World Food Programme (WFP) and a wide range of other international partners from developing countries, with the objective to catalyse actions to move undernutrition toward the centre stage of international discourse [21].

The action oriented framework from a national discourse (key service providers, policy managers and health managers) is aimed to garner local and governmental support to improving stunting, wasting and anaemia, and reduce inequities- in a setting situated among the lowest 32 countries with such health indices [4].

A summary of the key elements of the framework is provided as a template and shown in Table 2.

**Table 2:** Framework for national nutrition action for reducing nutritional deprivations in ghanathrough a data-driven planning approach

National Action Required	Prioritized strategies
***Scale up support for nutrition programs and capacity development:***	• Ministry of Health and Ministry of Finance to collaborate to earmark funds and budget allocation for nutritional commodities at all levels • Integrated training plan and budget for Family Health Services including reproductive and child health, Nutrition and health promotion • Strengthen prioritization and coordination (at higher levels) and integration of services (at lower levels) for nutritional interventions. • Integrate nutrition into new born training package and orientation programmes for all frontline staff and newly appointed/posted staff to both public and private health facilities and maternities • Revise and implement pre-service curriculum to enhance the practical skills of frontline workers in nutrition services • Strengthen active surveillance on severe acute malnutrition within the communities and increase frequency and intensity as well as targeting of cases of severe acute malnutrition with social mobilization activities. • Task sharing/shifting to other health care workers who traditionally have not been involved in nutrition services to expand the base of coverage (e.g. physicians assistants, enrolled nurses and midwifes), especially in the District Hospitals • Capacity building (training and supervision) for frontline health staff nutrition counselling and support at all levels through provision of resources (counselling cards, child health record booklets, registers)
***Mobilize key stakeholders in an inclusive approach to ownership and demand for services***	• Leverage on existing community platforms for community based intervention (e.g. exclusive breastfeeding and infant and young child feeding) advocacy activities (faith based organizations, community groups, women groups etc.). • Increase community participation in nutritional intervention especially breastfeeding and young child feeding through targeting other family members with decision making in breastfeeding (in-laws, grandmothers, peers, husbands) • Community dialogue meetings/sensitization, targeting key family and community stakeholders (Traditional, Religious and opinion leaders) on effects of social norms on nutrition and health of the young child • Engage Community Health Committees and other community based organizations as well as targeting family, traditional and religious leaders to create demand for Focus antenatal services where the women will receive the supplements- Iron, Folate and Vitamin A Supplementation
***Provide guide to co-ordinate work of stakeholders and partners***	• Develop and implement uniform data collection forms and registers for use in all health facilities and by partners • National and regional levels to develop factsheets/key messages on complementary feeding, educational materials on control of anaemia and micronutrient deficiencies for frontline health workers and community based volunteers. • Regular engagement of partners to streamline activities at national and sub-national levels, during health summit and review meetings.
**Develop strong, prioritised national strategies**	• Create a simplified register and incorporate essential nutrition indicators (Iron, Folate and Vitamin A Supplementation) into routine health information reporting system (DHIMS) to strengthen service monitoring • Review current social mobilization strategy on severe acute malnutrition to specifically include essential elements such as diet frequency, target groups, inimical social norms, and specific workable service delivery platforms. • Strengthen linkages and communication between health workers within health facilities and volunteers working at and within the communities- to engender prompt referrals and enrolment into care for children with severe acute malnutrition • Work schedule re-organization and task sharing at the health facility level to free time for sufficient and effective nutritional counselling by frontline workers. • Strengthen inter-sectoral collaboration- Ministry of Health and Ministry of Agriculture to increase involvement of agriculture extension in family or community farming activities
***Pay attention to inequities and special needs regions and geographical areas***	• Strengthen data validation, monitoring and supervision of frontline workers (Community health workers) at the decentralized level especially in regions and geographical areas with worse indicators • District health teams to be tasked and appraised on coordination and management of malnutrition services at the district and sub-district level • Health facility managers to enforce the implementation of the policy on free treatment for Children under-5 years at all levels of the health system to engender service uptake in poor rural areas of the country.
***Support with evidence, demonstrable gains in service delivery***	• Good quality data are essential for effective planning and analysis, and data validation to improve data quality and accuracy is a core activity to be undertaken by all levels • Build capacity of regional, district and sub-district on the application of data driven planning tools (Bottleneck Analysis/Determinants Analysis Approach is one means of achieving this) • Create supportive environment for nutritional surveys (national and sub-national level) with participation of partners. Hitherto, nutritional surveys have been limited to the national level.
***Support advocacy for addressing undernutrition:***	• Advocacy for improvement of Early Initiation of Breastfeeding in both public and private health facilities and maternities • Advocacy with National health insurance authority to adopt Baby Friendly Health Facility Initiative as one of the criteria for accreditation of health facilities in the country. • Advocacy at the national level for the application of E-platforms for teaching and learning in nutrition by pre-service institutions to enhance efficiency and quality of training

### Key lessons learned

Good quality data are essential for effective planning and analysis, and data validation to improve data quality and accuracy is a core activity that should be undertaken by all levels of the health system. The Determinant Analysis process enables health managers and frontline health workers to readily identify gaps, undertake a causal analysis of the barriers and identify solutions. It garners a data driven approach to implementation even at the lowest level.

### Study limitation

The purpose of the study was to assess nutrition services and therefore role of service providers at the basic level (street level bureaucrats) is essential and should have been involved in the assessment. However, critical issues from the basic level were captured by regional officers who work directly with these street level bureaucrats. In addition, the Ten Determinant Model used for the assessment did not explicitly capture indicators on policy, legal, social norms and budget-related factors that shape the determinants of health service coverage. However, these cross-cutting factors were systematically considered as part of analyzing each identified bottleneck during the causal analysis.

## Conclusion

Overall in Ghana, there were gaps and insufficient coverage for the six Lancet nutrition specific interventions assessed. Inadequate pre-service and in-service training for staff, low prioritization and coordination (at higher levels) and weak integration of services (at lower levels) were key barriers to nutrition coverage in Ghana. Data driven analysis and planning based on proven nutritional interventions demonstrated gaps and barriers and garnered workable strategies (framework to guide implementation) to improve nutrition services.

Specific recommendations for improving nutrition services include, districts, sub-districts and community level systems being supported with basic commodities to provide the full complement of services. Involvement of all health care workers at districts, sub-districts and community levels and community based volunteers, as well as involvement of health facility managers, stakeholders and partners will be key. Dissemination of all existing and new policies and field guides to the lower levels will be essential. National and sub-national levels should improve data validation activities through regular monitoring and coordination.

## Abbreviations

CBV: Community based volunteers
CHWs: Community Health Workers
CHPS: Community-based Health Planning Services
DHIMS: District Health Information Management System
EBF: Exclusive breast feeding
IFA: Iron and Folate Supplementation
IYCY: Infant and young child feeding
SAM: Severe acute malnutrition
UNICEF: United Nations Children Fund
VAS: Vitamin A Supplementation
WFP: World Food Programme
WHO: World Health Organization
